# Synbiotic Intervention Ameliorates Oxidative Stress and Gut Permeability in an In Vitro and In Vivo Model of Ethanol-Induced Intestinal Dysbiosis

**DOI:** 10.3390/biomedicines10123285

**Published:** 2022-12-19

**Authors:** Dhara Patel, Chirayu Desai, Deepmala Singh, Virupakshi Soppina, Kirti Parwani, Farhin Patel, Palash Mandal

**Affiliations:** 1 P. D. Patel Institute of Applied Sciences, Charotar University of Science and Technology, Nadiad 388421, Gujarat, India; dharapatel.phd@gmail.com (D.P.); kirtiparwani@gmail.com (K.P.); farhinpatel1993@gmail.com (F.P.); 2 Department of Environmental Biotechnology, Gujarat Biotechnology University, Gandhinagar 382355, Gujarat, India; chirayudesai@gbu.edu.in; 3 Indian Institute of Technology, Gandhinagar 382355, Gujarat, India; deepmala_19310008@iitgn.ac.in (D.S.); vsoppina@iitgn.ac.in (V.S.)

**Keywords:** gut microbiome, liver disease, synbiotic, combinational therapy

## Abstract

Alcoholic liver disease (ALD) alters gut microbiota and tight junctions, causing bacterial components to enter the portal vein and induce oxidative stress-induced inflammation in the liver. Only corticosteroids and liver transplants are treatment options for severe alcoholic hepatitis. ALD’s pathophysiology is unknown. However, acetaldehyde’s toxic effects cause oxidative stress and intestinal permeability. This study investigates the influence of a synbiotic (a combination of aged garlic extract (AGE) and *Lactobacillus rhamnosus* MTCC1423) on colonic oxidative stress and inflammation in ALD male Wistar rats and Caco2 cells. MDA measurement by HPLC in CaCo2 cells, blood serum, and colon tissue demonstrated that synbiotic treatment in the ALD model reduces oxidative stress. Further, fecal high-throughput 16S rRNA gene sequencing revealed the microbiome’s shift towards Firmicutes in the synbiotic group compared to ethanol. In addition, DCFDA labeling and H/E staining demonstrate that the synbiotic is beneficial in inhibiting the development of ALD. In the colon, the synbiotic reduces the activation of CYP2E1 and the inflammatory markers TNF-a and IL-6 while elevating the mRNA expression of ZO-1, occludin, and IL-10. Synbiotics colonize *Lactobacillus* to restore barrier function and microbiota and reduce colon oxidative stress. Thus, a synbiotic combination can be used in ALD treatment.

## 1. Introduction

Alcohol overuse causes three million deaths globally, more significant than mortality caused by tuberculosis, HIV/AIDS, and diabetes [1]. The ALD spectrum ranges from hepatic steatosis to hepatitis fibrosis, cirrhosis and carcinoma. The pathophysiology of ALD and the interorgan interaction between the liver and colon remain unclear as alcohol-related organ damage varies from person to person and is unpredictable. Given the clinical adverse effects, including inhibition of liver regeneration and bacterial infection, it is imperative to find novel medications capable of preventing and treating ALD and its related condition, as there is no U.S. food and drug administration (USFDA) approved medicine for the treatment of ALD other than sobriety.

A substantial amount of literature has shown that the development and progression of ALD are influenced by the gut–liver axis, which affects intestinal immune response and function and hepatic and systemic oxidative stress and inflammation. Through portal circulation and close bidirectional communication between the gut environment and the liver during metabolism, the liver is intimately connected to the intestine [2]. Recent research demonstrates that chronic alcohol intake affects the gut tight junctions (TJs), resulting in increased gut permeability, which is associated with the progression of ALD [3]. TJs such as occludin, claudins, and zonula occludens (ZO) modulate intestinal mucosal permeability; thus, regulating the expression of TJs will have a beneficial impact on the permeability of the intestine in ALD [4,5]. Alcohol increases intestinal permeability, which causes bacterial endotoxins to move into the portal vein and cause liver inflammation [6]. According to clinical studies, alcoholics’ elevated blood endotoxemia may be caused by gut dysbiosis or gut leakiness [7,8]. The Gram-negative bacterial cell wall component lipopolysaccharide (LPS) engages an innate pro-inflammatory immune system. It activates the NF-κB pathway, a vital link in the gut–liver interaction [9,10]. Therefore, it is hypothesized that gut permeability and altered gut barrier function play a role in developing systemic inflammation in alcohol-induced hepatotoxicity [11].

The precise molecular mechanism through which alcohol increases intestinal permeability is unclear. According to a study, the integrity of the gut barrier is compromised by oxidative stress to the cytoskeleton in the presence of alcohol [12]. CYP2E1 is an alcohol-metabolizing enzyme that contributes to the gastrointestinal effects of the alcohol [13]. The modification of intestinal permeability may be mediated by stimulation of CYP2E1 metabolism, formation of reactive oxygen species (ROS), and oxidative stress [14]. Moreover, ROS can be exacerbated by bacterial translocation and pro-inflammatory cytokines leading to oxidative stress in the gut.

Additionally, alcohol can increase lipid synthesis, leading to excess lipid accumulation. Increases in hepatic lipid metabolism may be influenced by oxidative stress [15]. As a result, variables contributing to the onset and progression of ALD include oxidative stress, lipid accumulation, inflammation, increased intestinal permeability, and imbalanced gut flora.

The gut microbiota or microbial community is essential in ALD therapy or prevalence [16]. A healthy gut microbiota affects intestinal architecture, immunity, and gut barrier function [16,17]. Chronic alcohol use causes dysbiosis, related to decreased *Lactobacillus* spp. and increased *Enterobacteriaceae* [18]. Prebiotic, probiotic, and synbiotic administration may reduce alcohol-induced gastrointestinal damage. Probiotics and prebiotics are used as functional foods to prevent alcohol-induced changes in colonic epithelial TJs and barrier failure [19]. In vivo study suggests that *Lactobacillus rhamnosus* GG (LGG) reduces intestinal inflammation, oxidative stress, and gut leakiness [20].

Pharmacotherapeutic investigations have revealed that garlic’s sulfur-containing bioactive component is a promising target for developing functional components in staple foods and novel therapeutics due to its considerable resources and non-toxic nature. Garlic possesses antioxidant, anticancer, immunomodulatory, hypolipidemic, and organ-protective effects. The aging of garlic increases its properties, as mentioned earlier. Recent studies have reported that AGE may prevent ALD due to its anti-inflammatory effects [21]. AGE reduces intestinal dysbiosis. AGE helps stabilize the gut flora in nonalcoholic fatty liver disease (NAFLD) [22]. Garlic protects rats from chronic and acute ethanol-induced liver damage by an antioxidant mechanism [23]. AGE increases TJ function and decreases oxidative stress and inflammatory responses in LPS-stimulated 3T3-L1 cells and rats [24,25]. However, the implications of AGE on gut microbiota, integrity, and pathways remain unclear. Therefore, it is essential to investigate the potential mechanisms of this synbiotic as a combination therapy for the prevention and treatment of alcohol-related liver disease and intestinal microbiota.

The present work aims to investigate the potential mechanism of a synbiotic against chronic ALD by examining colon barrier integrity, oxidative stress, and inflammation in epithelial colon cells (Caco-2) and alcohol-induced colon injury in male Wistar rats. This study can provide a basis for comprehending the molecular process that promotes gut flora in the presence of alcohol, leading to more effective ALD prevention and treatment.

## 2. Materials and Methods

### 2.1. Microbial Culture Collection and Chemicals

Kyolic (Mission Viejo, CA, USA) provided the aged garlic extract. MTCC 1423 *Lactobacillus rhamnosus* was acquired from MTCC in India. Sigma-Aldrich (St. Louis, MO, USA) provided the iso-butyl methylxanthine and Tri reagent (Sigma-Aldrich, CA, USA). Gibco provided the fetal bovine serum (FBS) (Sigma-Aldrich, CA, USA). Applied Biosystems delivered the kits for cDNA synthesis and the SYBR green master mix (Sigma-Aldrich, CA, USA). Sigma provided commercially produced primer sequences. HiMedia Laboratories provided Molecular Biology grade ethanol with a purity of at least 99.8% and other chemicals (Mumbai, Maharashtra, India).

### 2.2. Culturing of Caco-2 Cell Line

In this investigation, Caco-2 cells, a colonic epithelial cell line, were provided by the National Centre for Cell Sciences (NCCS), India. Caco-2 was maintained in Dulbecco’s modified eagle medium (DMEM) complete media as per the protocol mentioned in Farhin et al. [26]. The investigational dose of *Lactobacillus rhamnosus* MTCC1423 and AGE was 10^9^ CFU/mL of cell-free supernatant and 10 μL of 100 mM ethanol. For each experiment, passages 31 to 39 of the cells were tested.

### 2.3. Caco-2 Cells Experimental Dosing for Chronic Ethanol Model

*L. rhamnosus* MTCC1423 was cultivated at 37 °C to 10^9^ CFU/mL in an MRS medium. The cell pellet was obtained per the protocol mentioned in Farhin et al., 2021, and the cytosolic fraction of 10^8^ CFU/mL cells resuspended in nuclease-free water was used for the experiment ts stockpiled at −20 °C for additional experimentation.

### 2.4. Animals Housing and Chronic Ethanol Feeding of Male Wistar Rats

Zydus Pharmaceutical Industries Pvt. Ltd. supplied eight-week-old, 200–225 g male Wistar rats (India). The protocol involving the use of animals for the study was approved by the Institutional Animal Ethics Committee of Ramanbhai Patel College of Pharmacy, Charotar University of Science and Technology (RPCP/IAEC/2021-22/R14). Rachdaoui, N. et al. previously detailed the chronic ethanol feeding technique employed in the study [27]. After the rats were obtained, they had unrestricted access to a standard chow diet and water for three days to acclimatize them. Later, they were acclimatized with the liquid Lieber–DeCarli diet for four days before the initiation of the experiment.

### 2.5. Establishment of Intestinal Barrier Dysfunction with Ethanol and Analysis of the Preventive Effect of Probiotics and AGE Administration

#### 2.5.1. In Vitro and In Vivo Experimentation

In vitro research used four sets of Caco-2 cells. The monolayers were assessed after 1.3 × 10^5^ cells/mL were seeded in 6 wells plates. In group A, the cells were with no treatment; in group B, they were administered with 100 mM ethanol serum-free medium to induce intestinal epithelial barrier dysfunction. Group C: cells were treated with 10 μL/mL AGE solution for 24 h. Group D: cells were supplemented with serum-free media constituting 10 μL/mL AGE and 10^9^ CFU cytosolic fraction, and 100 mM ethanol.

The rats were fed Lieber–DeCarli for two days to acclimatize them. After acclimatization, rats had unrestricted access to 1% ethanol-Lieber–DeCarli food. The ethanol-stimulated intestinal barrier damage trial lasted 25 days with increasing ethanol concentrations (*v*/*v*) as described in Mutlu, E. et al., 2009 [26]. The synbiotic was administered orally at 109 CFU/ day *Lactobacillus rhamnosus* MTCC 1423 and 200 mg/kg of AGE to the respective rat’s group. At the end of the feeding trial, i.e., 5 weeks, fecal samples were collected from the rats of each group under 12 h fasting condition. Later, fasting rats were anesthetized with Pentobarbital (Nembutal^®^) 40–60 mg/kg, followed by blood withdrawal from the vena cava. Colon specimens were later collected, fixed in RNA, and processed per the protocol described in Kema et al., 2017 [28]. Serum and fecal samples were stored at −80 °C until further use.

To investigate the gut barrier damage, rats (N = 6) were grouped as follows: (A) the control: pellet fed rats; (B) the ALD group: rats were given the Lieber–DeCarli liquid food; (C) the preventative therapy group 1: rats were supplemented with 200 mg/kg AGE and ethanol; (D) the preventative therapy group 2: rats were supplemented with 10^9^ CFU/day *Lactobacillus rhamnosus* MTCC 1423 and 200 mg/Kg AGE with ethanol. Both preventative therapies were given simultaneously with the Lieber–DeCarli food for 3.5 weeks to induce gut dysfunction.

### 2.6. Caco-2 Transepithelial Electrical Resistance (TEER) Evaluation

Caco-2 was seeded at 76,000 cells/cm^2^/transwell and was used to evaluate intestinal barrier integrity. TEER was assessed based on the groups indicated in Section 2.5.1. A total of 0.5 mL media was applied to the upper chamber, and 1.5 mL to the bottom chamber section and helical volt ohmmeters measured TEER. Inserts devoid of cells were designated blanks, and the mean resistance of cells was removed from samples of control and treatment. Electrical resistance was calculated by conducting the experiment three times for each monolayer and averaging the results [27].

### 2.7. Hematoxylin and Eosin (H/E) Colon Staining

Formalin-fixed, OCT-treated colon sections; cryostats cut 5–10 μM colon sections and stained them with hematoxylin-eosin (H/E) to distinguish morphological changes. Blinded researchers examined all the slide sections.

### 2.8. Lipogenesis Analysis by Oil Red O Staining Confocal Imaging

The colon tissue samples were stained with Oil red O mounted on the glass slide. The samples were imaged using a Leica laser scanning confocal microscope (TCSSPI8). The Oil red O dye was imaged with a 561 nm laser at 0.3%, and a HyD detector was used at 80% gain to collect emission from the dye in the range 573–669 nm. The images were captured in 1024 × 1024 format with a 100 Hz speed.

### 2.9. Caco-2 Cells ROS Estimation

Caco-2 cells were treated for 24 h with respective groups, as mentioned in Section 2.5.1. A DCFDA/H2DCFDA-cellular ROS Assay Kit (Abcam, ab113851, USA) was used to measure intracellular ROS [24]. Plate reader analysis estimated fluorescence intensities.

### 2.10. Colon Oxidative Stress Assessment

#### 2.10.1. Colonic Malondialdehyde (MDA) by TBA Method

A total of 500 mg of fresh colon tissue was homogenized at 24,000 rpm/min with 1.15 percent KCl. After combining the homogenate with 250 uL of 6M NaOH and incubating at 67 °C for 43 min, the mixture was vortexed with the same amount of acetonitrile. When the samples had been hydrolyzed, they were centrifuged for ten minutes at a speed of 15,000× *g*. The supernatant (240 μL) was combined with 2,4-dinitrophenylhydrazine (DNPH) (24 μL). For another 10 min, it was kept in the dark. The resultant samples were evaluated by HPLC (Waters Breeze-2, USA) via the ODS2 reverse phase column (Waters Breeze-2, USA). The mobile phase was 38: 62 acetonitrile: 0.2% acetic acid HPLC grade water. MDA in the sample was measured at 310 nm using isocratic HPLC and a UV detector. As per procedure, a standard curve was generated using 20 nmol/mL of MDA standard solution (TCI, Japan) diluted with 1% H_2_SO_4_ [26,29,30].

#### 2.10.2. HPLC Method for Serum MDA Content Evaluation

A total of 500 μL serum was combined with 100 μL of 6M NaOH at 60 °C for 40 min. For acidification, hydrolyzed serums were mixed with 35% perchloric acid and centrifuged for 10 min at 37 °C. Subsequently, for 10 min, 250 μL of supernatant was incorporated with 25 μL of 2,4-dinitrophenylhydrazine (DNPH). A serum sample that had been derivatized was analyzed using an HPLC (Waters Breeze-2, USA) with an ODS2 reverse-phase column. The mobile phase was 38: 62 acetonitrile: 0.2% acetic acid HPLC-grade water. MDA concentration in serums was measured at 310 nm using HPLC under isocratic conditions [29].

#### 2.10.3. RNA Extraction and qRT-PCR

In 6-well plates, Caco-2 cells were seeded (2.3 × 10^5^ cells/mL) and cultivated for three weeks. After 24 h, 10 μL AGE (1 mM), *Lactobacillus rhamnosus* MTCC 1423 (10^9^ CFU/mL), and ethanol (100 mM) were supplemented, and RNA was extracted from cells using the TRIzol method. Formaldehyde gels were used for quality control analysis. In vivo investigations used TRIzol Reagent to extract 4 mg of colonic RNA. NanoDrop (Thermo Fisher Scientific, Waltham, MA, USA) measured concentrations of RNA in ng/ul and purity (A260/A280). Total RNA was processed with DNase, and cDNA was synthesized using a cDNA synthesis kit, as shown in the manufacturer’s instructions. The primers listed in Table 1 and Table 2 were used. Utilizing an Agilent Mx3005P qRT-PCR system (Agilent Stratagene, Hyderabad, India) and SYBR/ROX Master Mix, gene expressions were generated. The 18S rRNA gene was used as an endogenous control. Results were shown as fold-over changes in basal level compared to the control group [31].

### 2.11. Analytical Statistics

Mean −/+ SD was determined for N = 6 replicates. For statistical analysis, GraphPad Prism 7 and one-way ANOVA in conjunction with the Tukey post hoc test were calculated. When associated with the ethanol group, significance (*p*-value) is defined as * *p* < 0.05, ** *p* < 0.01, *** *p* < 0.001, **** *p* < 0.0001.

### 2.12. Feces Collection

After the experiment, fresh samples were gathered and put in sterilized tubes before being deposited in a freezer at −80 °C for future investigation.

#### High-Throughput 16S rRNA Gene Amplicon Sequencing

A DNA extraction kit collected 500 mg of fecal bacterial DNA (HiPurA Genomic DNA Purification Kit, Himedia, India). DNA concentration was evaluated using NanoDrop™ 2000 (Thermo Fischer, Waltham, MA, USA). For PCR amplification, 50 ng of DNA was used. Eurofins Genomics, Bengaluru, amplified the 16S rRNA gene (V3-V4 region) and prepared and sequenced libraries using the Illumina MiSeq platform. FASTQ files containing de-multiplexed 2300-bp paired-end readings were assessed using Quantitative Insights into Microbial Ecology 2 (QIIME 2). The “demux summarise” plugin was used to evaluate paired-end read quality, and the “dada2 denoise-paired” plugin was used to denoise, and filter sequence reads. The sequence reads were taxonomized using the “feature classifier (classifier-sklearn)” plugin, a pre-trained naive Bayesian classifier, and the latest edition of the SILVA database (Silva 138, 99 percent OTUs from 515F/806R region of sequences). The taxonomy was shown using QIIME2-view’s “taxa barplot” plugin per the procedure described in Patel et al., 2021 [32].

## 3. Results

### 3.1. Synbiotic Intervention Lowers Morphological and Intestinal Barrier Alterations in Caco-2 Cells

**Figure 1:**
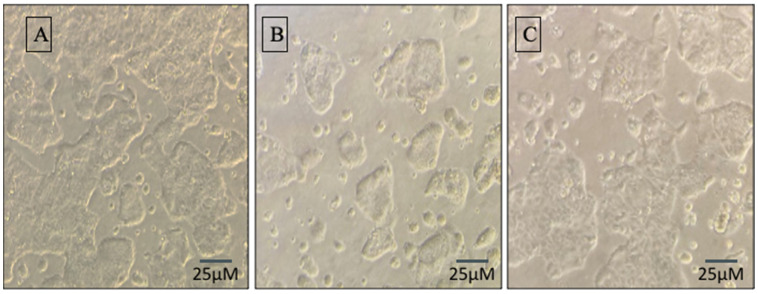
The appearance of Caco-2 cells is enhanced by synbiotic preventive therapy: Caco-2 cells were given the following dosage: (**A**) control, (**B**) ALD model: 100 mM ethanol, (**C**) synbiotic (100 mM + 10 g/mL AGE+ 10^9^ CFU/mL cell-free supernatant) and 100 mM ethanol and restored the morphology of cells. All pictures were captured at an ×100.

Caco-2 cells exhibit healthy patches of cells, as does the intestinal cell line, but the patches are fragmented and smaller in the cells with ethanol dose. Synbiotic therapy improves cell growth patterns in cell patch images resembling control cells (Figure 1).

**Figure 2:**
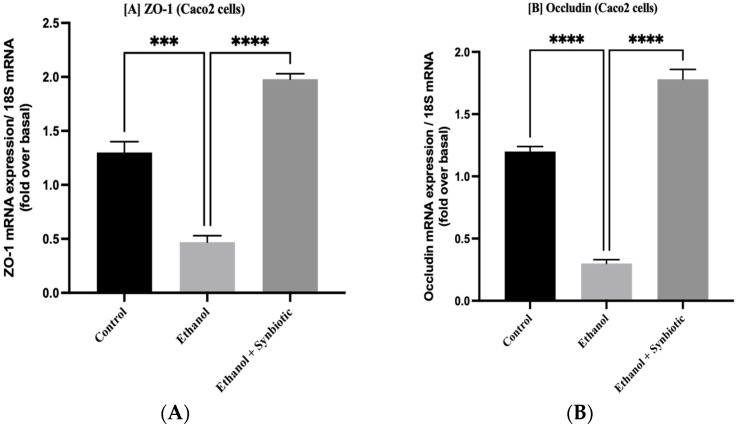

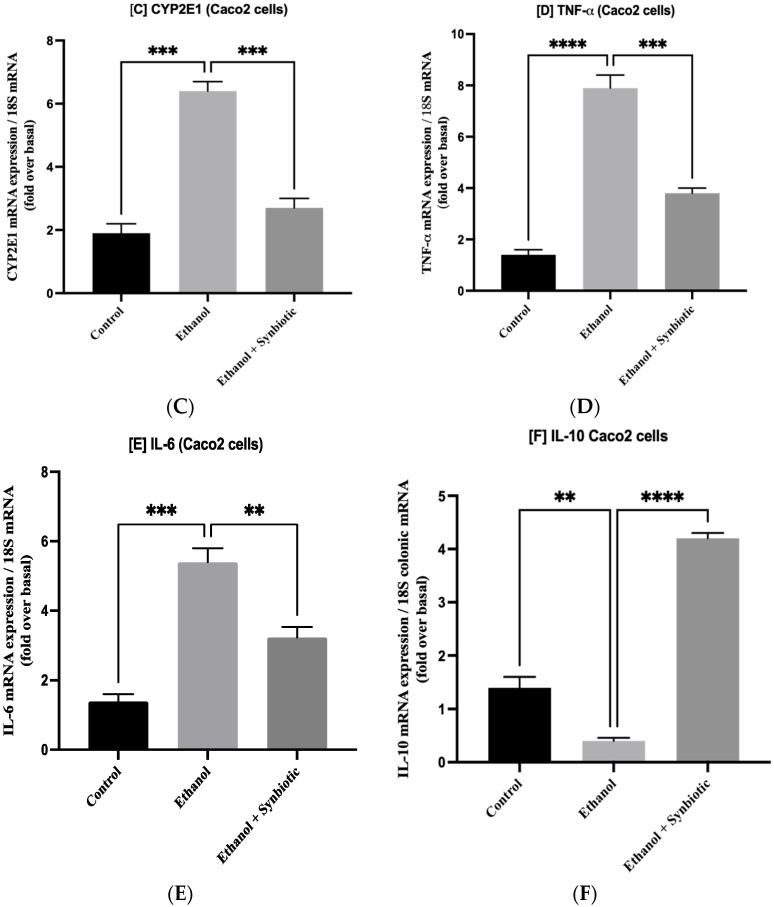
The effects of AGE and synbiotic on Caco-2 cells treated with ethanol. Tight junctions (**A**) ZO-1 and (**B**) occludin mRNA expression, as well as presentation of markers of inflammation (**C**) TNF-a, (**D**) CYP2E1, (**E**) IL-6, and anti-inflammatory indicator (**F**) IL-10, are all reported. The statistical analysis was calculated as mentioned in Section 2.11. Mean −/+ SD was determined for N = 6 replicates. For statistical analysis, GraphPad Prism 7 and one-way ANOVA in conjunction with the Tukey post hoc test were calculated. When associated with the ethanol group, significance (*p*-value) is defined as ** *p* < 0.01, *** *p* < 0.001, **** *p* < 0.0001.

The ethanol group exhibits reduced mRNA expression of tight junctions (TJ) such as occludin and ZO-1 and anti-inflammatory marker IL-10 compared to the control. Inflammatory indicators such as CYP2E1, IL-6 and TNF-α are higher in Caco-2 cells treated with alcohol, contributing to intestinal permeability in the ALD model. Figure 2 shows that synbiotic treatment enhances TJ proteins and lowers markers of inflammation in Caco-2 cells more than AGE individually.

#### TEER Analysis

**Figure 3:**
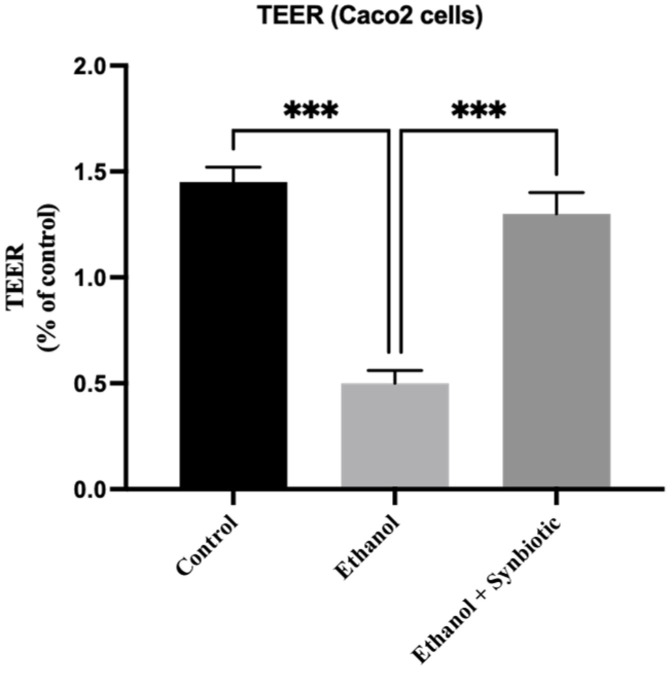
TEER assessment of Caco-2 integrity: TEER investigation of the impact of probiotics and AGE individually or as synbiotic on the functionality of the intestinal barrier produced by ethanol in monolayers of Caco-2 cells. The statistical analysis was calculated as mentioned in Section 2.10. Mean −/+ SD was determined for N = 6 replicates. For statistical analysis, GraphPad Prism 7 and one-way ANOVA in conjunction with the Tukey post hoc test were calculated. When associated with the ethanol group, significance (*p*-value) is defined as *** *p* < 0.001.

Ethanol reduced TEER in an in vitro model. Figure 3 shows that synbiotic administration to ethanol-exposed cells increased TEER substantially. These results suggest that ethanol impairment to intestinal barrier function may be restored in both in vitro and in vivo models.

### 3.2. Synbiotic Improves Weight and Length Proportion of Colon in ALD Rats

**Figure 4:**
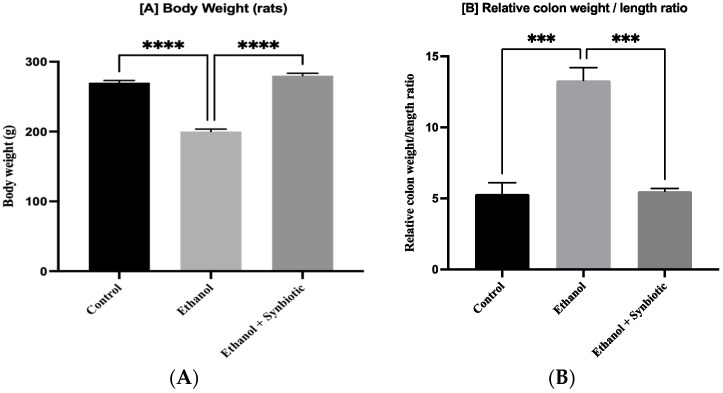
Synbiotic (AGE + *L. rhamnosus*) affects gut dysbiosis caused by ethanol dosage. (**A**) Body weight and (**B**) relative weight and length proportion of the colon are represented above. The statistical analysis was calculated as mentioned in Section 2.10. Mean −/+ SD was determined for N = 6 replicates. For statistical analysis, GraphPad Prism 7 and one-way ANOVA in conjunction with the Tukey post hoc test were calculated. When associated with the ethanol group, significance (*p*-value) is defined as *** *p* < 0.001, **** *p* < 0.0001.

The alcohol group had lower BW and a more significant colon weight/length ratio than the control animals. Figure 4 demonstrates that synbiotic treatment enhanced overall weight and reduced the intestinal weight and length proportion compared to an ethanol group.

### 3.3. Synbiotic Enhances Rat Colon Tissue Integrity

**Figure 5:**
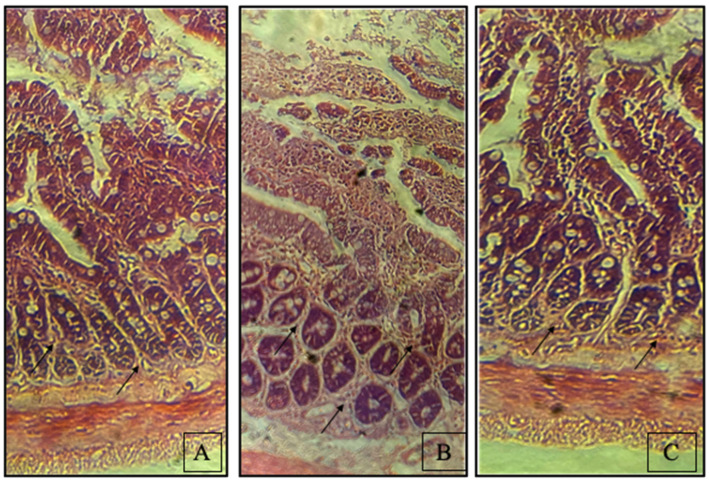
The influence of the synbiotic on the reduction of ethanol-mediated inflammation in the colonic epithelium as assessed by histopathological changes. The colon of a rat in its raw state (100×). These are the microscopic images: (**A**) control rats (**B**) rats fed 100 mM ethanol in increasing amounts (**C**) rats given ethanol and synbiotic (10^9^ CFU/day *L. rhamnosus* and 200 mg/kg AGE).

The colon segments stained with H/E showed highly structured epithelial lining along with Lieberkühn crypts. Abnormal epithelium, distorted crypts, reduced Lieberkühn crypts and altered colon mucosa indicate that ethanol affected the gut barrier function. As demonstrated in Figure 5, the synbiotic-treated group had comparative histology to the control group.

### 3.4. Synbiotic Intervention Reduced Lipogenesis in the Rat Colonic Epithelium

**Figure 6:**
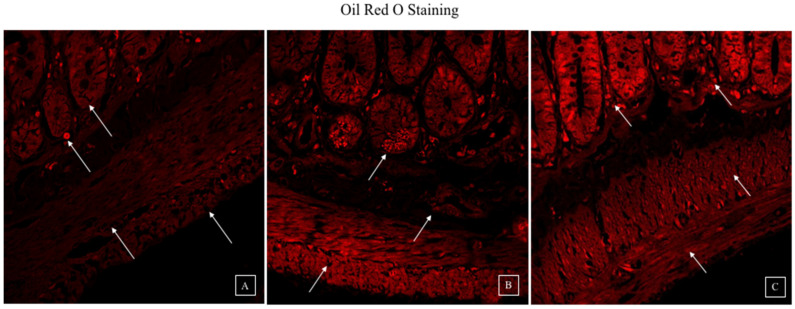
Confocal images of lipogenesis staining by Oil red O as standard and C2 stain is the novel lipid stain (**A**) control rats (**B**) rats exposed to 100 ethanol in increasing amounts (**C**) rats administrated with ethanol and synbiotic (10^9^ CFU/day *L. rhamnosus* MTCC1423 and 200 mg/kg AGE). The staining of lipid droplets (ethanol group), disruption of the colon epithelial layer in the presence of ethanol, and regaining of the epithelial barriers in the company of the two treatments (AGE and synbiotic) are represented by arrows.

The lipid staining of the colon bilayer with Oil red O dye revealed the development of lipid droplets associated with Lieberkühn crypts and an atypical shape indicated by the disruption of TJs in the ethanol group. As illustrated in Figure 6, the synbiotic intervention reduced lipogenesis and improved the epithelial barrier compared to the control group.

### 3.5. Synbiotic Treatment Reduces the Effects of Alcohol on TJ Expression and Permeability in the Colon

**Figure 7:**
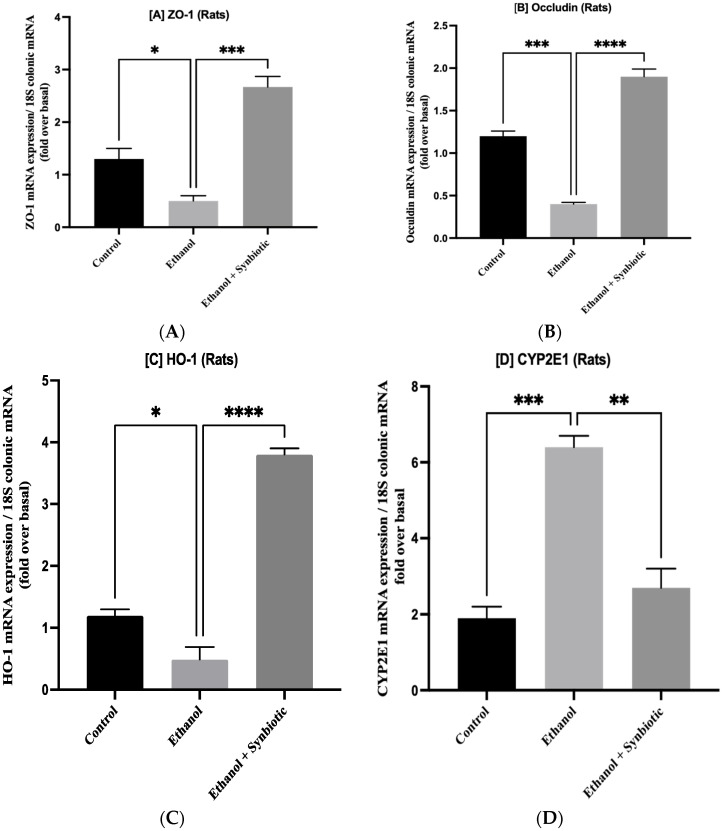

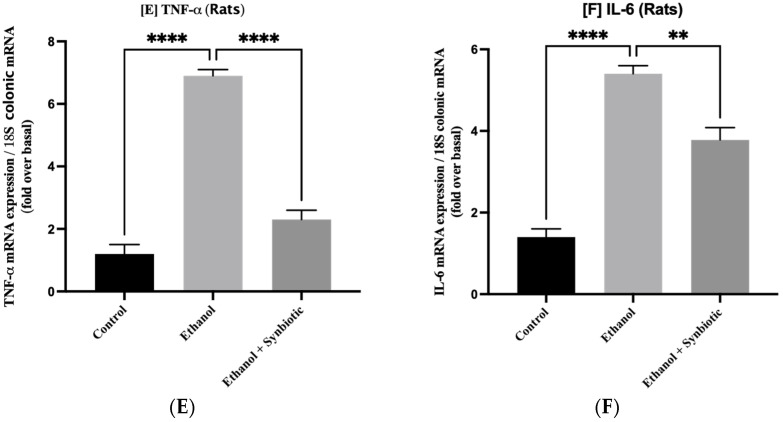
Synbiotic (AGE + *L. rhamnosus* MTCC 1423) mRNA expression on ethanol-induced gut dysbiosis. Expression of (**A**) ZO-1 and (**B**) occludin, (**C**) anti-inflammatory biomarker HO-1 as well as inflammatory biomarkers (**D**) CYP2E1, (**E**) TNF-a, and (**F**) IL-6. The statistical analysis was calculated as mentioned in Section 2.10. Mean −/+ SD was determined for N = 6 replicates. For statistical analysis, GraphPad Prism 7 and one-way ANOVA in conjunction with the Tukey post hoc test were calculated. When associated with the ethanol group, significance (*p*-value) is defined as * *p* < 0.05, ** *p* < 0.01, *** *p* < 0.001, **** *p* < 0.0001.

The transcription of ZO-1 and occludin in ethanol-treated Caco-2 monolayer cells and rat colon were significantly lowered, indicating that TJs are essential for gut health (Figure 6). Inflammatory indicators such as CYP2E1, IL-6, and TNF-a are reduced in the synbiotic group compared to the ethanol group, while enzyme-like heme-oxygenase (HO-1) regulates antioxidant machinery significantly. The anti-inflammatory mRNA levels of HO-1 genes in colonic mucosa were decreased by ethanol consumption. The synbiotic treatment considerably elevated the mRNA levels of HO-1 compared to the ethanol and AGE-fed rats (Figure 7). The correlation between in vitro and in vivo mRNA expression suggests that the synbiotic combination effectively lowers inflammation and increases tight junctions’ expression.

### 3.6. Preventative Therapy Synbiotic Reduces Ethanol-Mediated Intestinal Oxidative Stress

**Figure 8:**
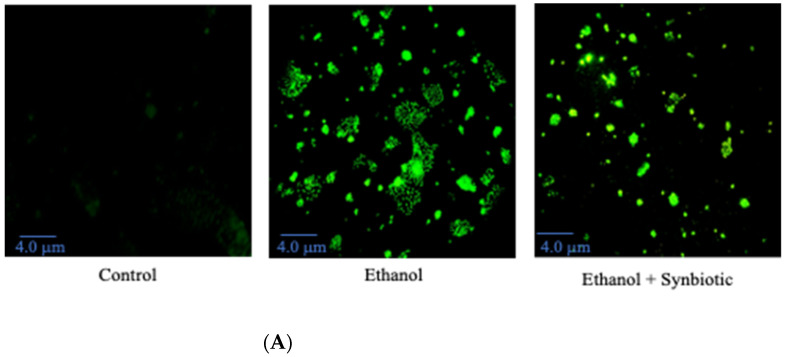

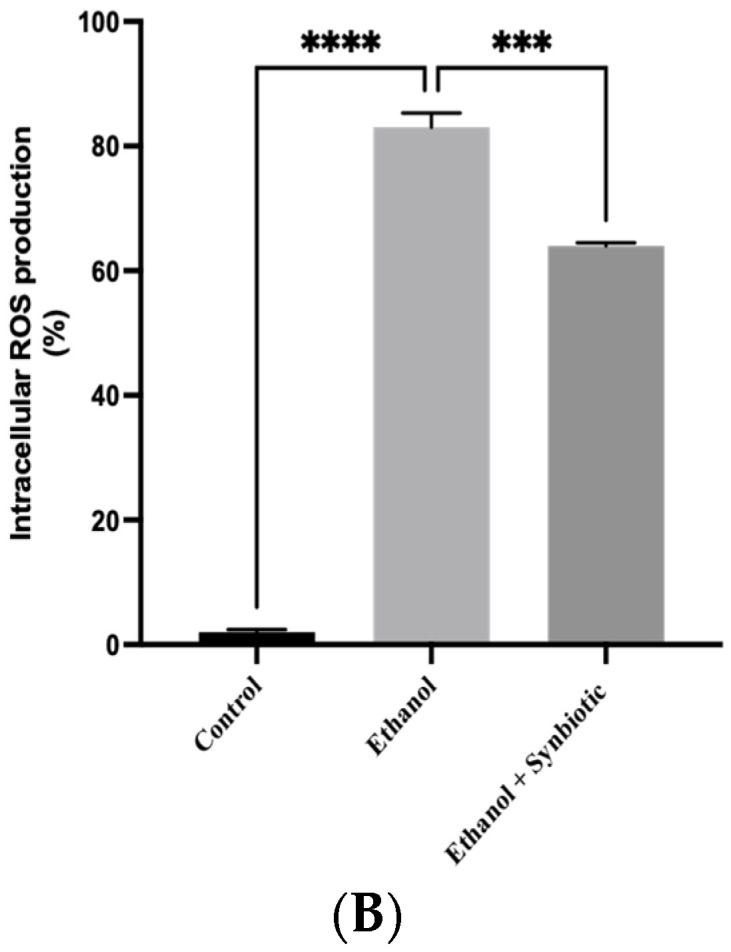
(**A**) Synbiotic (AGE + *Lactobacillus rhamnosus*) affects ethanol-induced intestinal dysbiosis when used alone or in combination. (**B**) Fluorescence staining and spectroscopy at 485/525 nm were conducted following carboxy-H2-DCFDA incubation. The scale of the images is 4.0 μm. The statistical analysis was calculated as mentioned in Section 2.10, *** *p* < 0.001, **** *p* < 0.0001.

**Figure 9:**
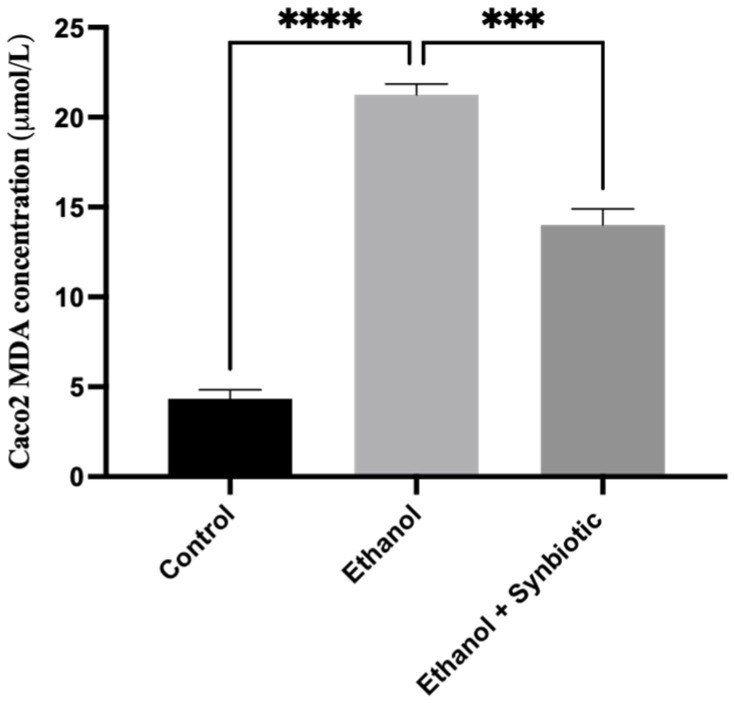
On Caco-2 cells, a graphical depiction of the synbiotic (AGE + *L. rhamnosus*) analysis of MDA concentration using the HPLC method. The statistical analysis was calculated as mentioned in Section 2.10, *** *p* < 0.001, **** *p* < 0.0001.

**Figure 10:**
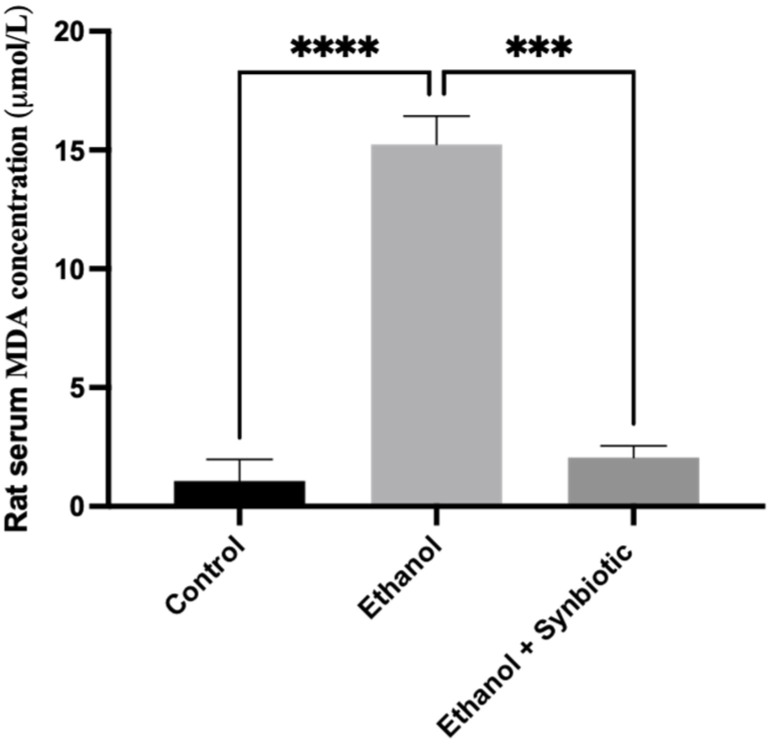
The HPLC technique determined the concentration of MDA in rat serum samples. The effect of the synbiotic (AGE + *Lactobacillus rhamnosus*) on gut dysbiosis is investigated in the presence of ethanol. Results reveal that synbiotic preventive therapy is more beneficial in treating ALD. The statistical analysis was calculated as mentioned in Section 2.10, *** *p* < 0.001, **** *p* < 0.0001.

We investigated the formation of reactive oxygen species (ROS) in Caco-2 cells and rat colon tissues to see how the synbiotic affected intestinal oxidative stress. We observed that ethanol stimulation enhanced ROS production; however, synbiotic therapy significantly lowered ROS accumulation compared to the ethanol group (Figure 7, Figure 8, Figure 9 and Figure 10). The production of Malondialdehyde (MDA) is increased due to the creation of ROS. Caco-2 cells had to decrease oxidative stress in the presence of the synbiotic, as seen in Figure 8. HPLC analysis was carried out with Caco-2 cells, rat serum, and colon tissue. The ethanol group had higher blood MDA levels than the control groups, corroborating earlier findings.

#### 3.6.1. High-Performance Liquid Chromatography (HPLC) for MDA Analysis of Caco-2 Cells

MDA levels in ethanol-treated Caco-2 monolayer cells were 20.03 ± 0.04 uM/L compared to 3.5 ± 0.05 μM/L in the control group. Figure 8 shows that Caco-2 with the synbiotic and ethanol had lower MDA levels of 14.6 ± 0.05 μM/L than the ethanol groups.

#### 3.6.2. HPLC Analysis of Rat Serum Samples

Compared to the control group (0.07 ± 01 μM/L), ethanol-treated rats had a serum MDA content of 15.03 ± 0.07 uM/L. However, as shown in Figure 9, the prophylactic therapy of synbiotics with ethanol significantly lowered MDA levels to 2.20 ± 0:02 uM/L.

#### 3.6.3. MDA Concentration of Rat Colon Tissue by HPLC Method

Compared to control rats (0.06 ± 0.01 uM/L), the ethanol group had 20.07 ± 0.06 uM/L MDA. Figure 10 shows that rats were given the synbiotic (10.46 ± 0.04 uM/L), indicating the decrease in oxidative stress in rat colon tissue in ALD.

### 3.7. Synbiotic Administration Reduces Rat Colonic Metabolism of Lipids

**Figure 11:**
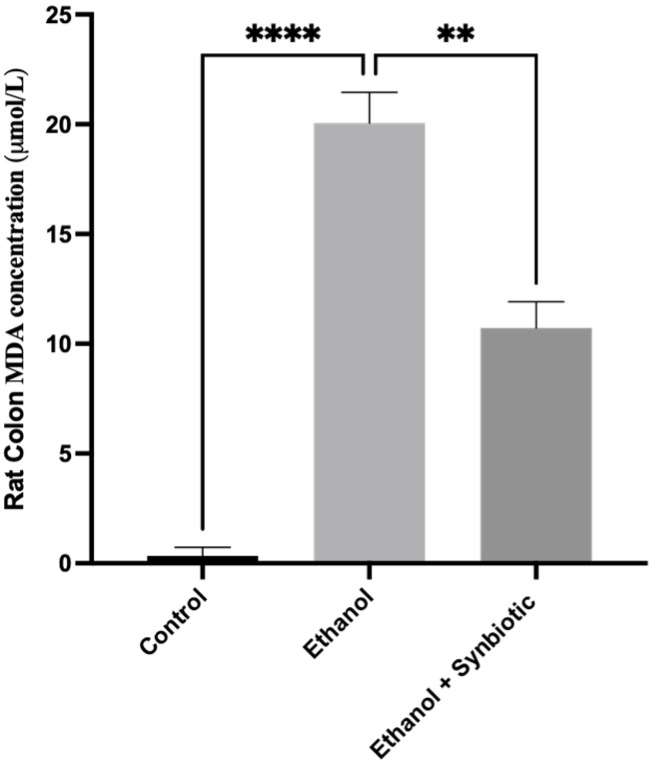
HPLC analysis of MDA concentration of rat colon tissue in synbiotic (AGE + *L. rhamnosus*) preventive treatment has a protective effect on rat colon tissue by lowering MDA concentration even in ethanol. The statistical analysis was calculated as mentioned in Section 2.10. Mean −/+ SD was determined for N = 6 replicates. For statistical analysis, GraphPad Prism 7 and one-way ANOVA in conjunction with the Tukey post hoc test were calculated. When associated with the ethanol group, significance (*p*-value) is defined as ** *p* < 0.01, **** *p* < 0.0001.

**Figure 12:**
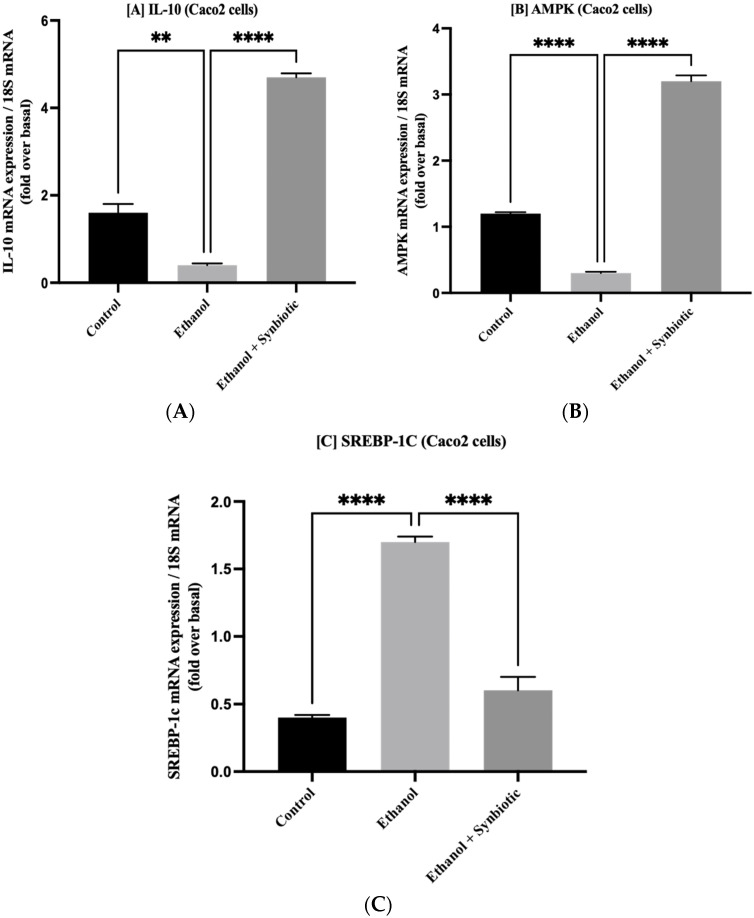
The mRNA expression of (**A**) IL-10, (**B**) AMPK, and (**C**) SREBP-1c in Caco2 cells is illustrated here. The anti-inflammatory markers IL-10 and AMPK were considerably increased after synbiotic (AGE + *L. rhamnosus*) treatment, whereas the inflammatory marker SREBP-1c was decreased. The statistical analysis was calculated as mentioned in Section 2.10. Mean −/+ SD was determined for N = 6 replicates. For statistical analysis, GraphPad Prism 7 and one-way ANOVA in conjunction with the Tukey post hoc test were calculated. When associated with the ethanol group, significance (*p*-value) is defined as ** *p* < 0.01, **** *p* < 0.0001.

Adenosine monophosphate-activated protein kinase (AMPK) concentrations were measured to assess the synbiotic effect on the intestinal barrier. Alcohol use promotes aberrant lipid metabolism in the colonic mucosa of ethanol-fed rats due to decreased AMPK activity, which enhances sterol regulatory element-binding protein 1c (SREBP-1c) levels and enhances lipogenesis. The synbiotic treatment increased AMPK levels. As seen in Figure 11, activation of AMPK suppresses ethanol-mediated lipogenesis by downregulating the transcription factor SREBP1c in the synbiotic group. Correlating the in vivo findings of Figure 11 with the Oil red O staining of Caco-2 cells indicated that ethanol-exposed cells deposited more lipids than control cells in Figure 12. These results reveal that the synbiotic may successfully suppress lipid accumulation in ethanol-treated Caco-2 cells and colon tissue.

### 3.8. High-Throughput 16S rRNA Gene Sequencing of Rat Feces Demonstrates That Synbiotics Restore Gut Flora

**Figure 13:**
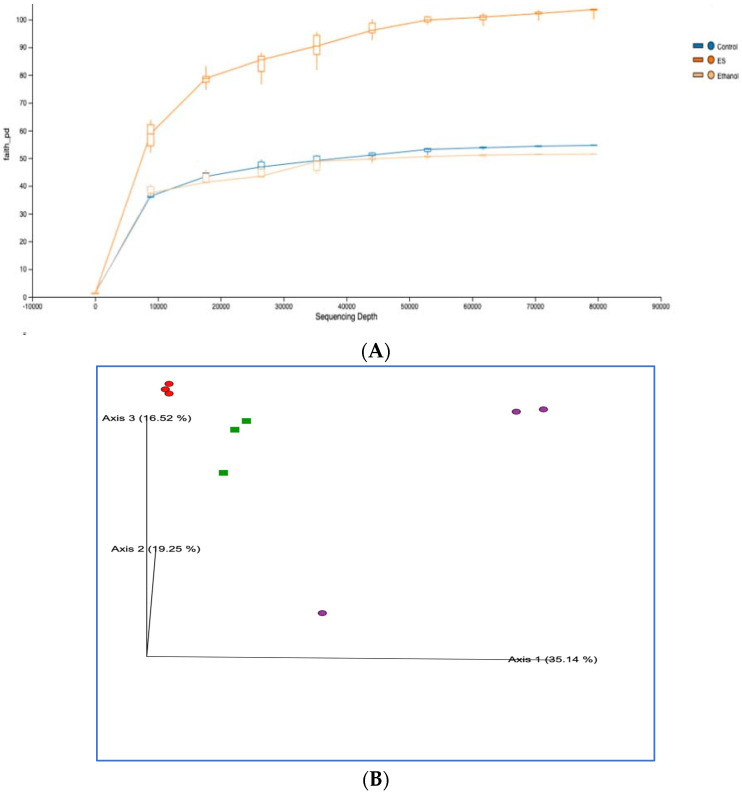

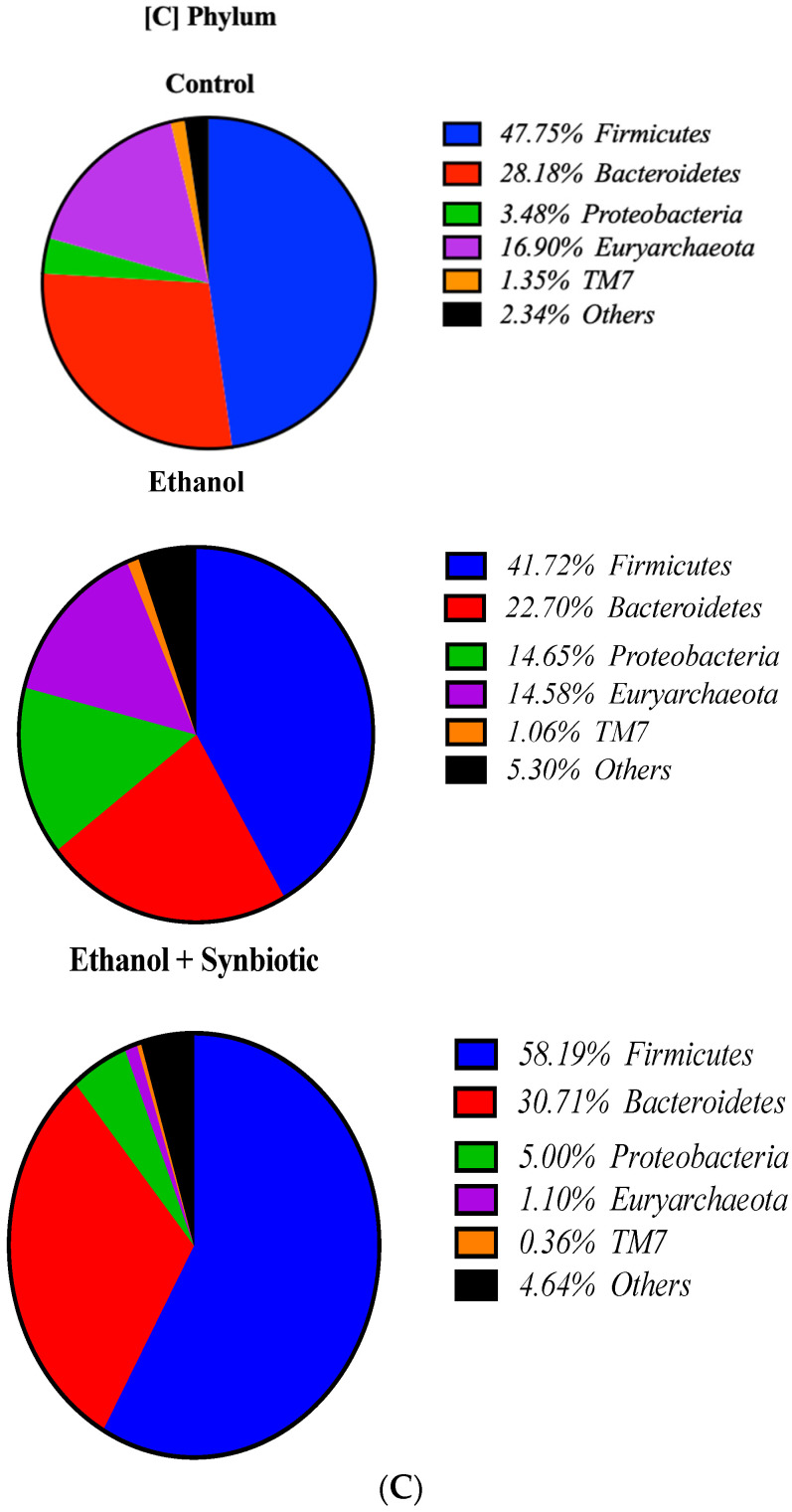

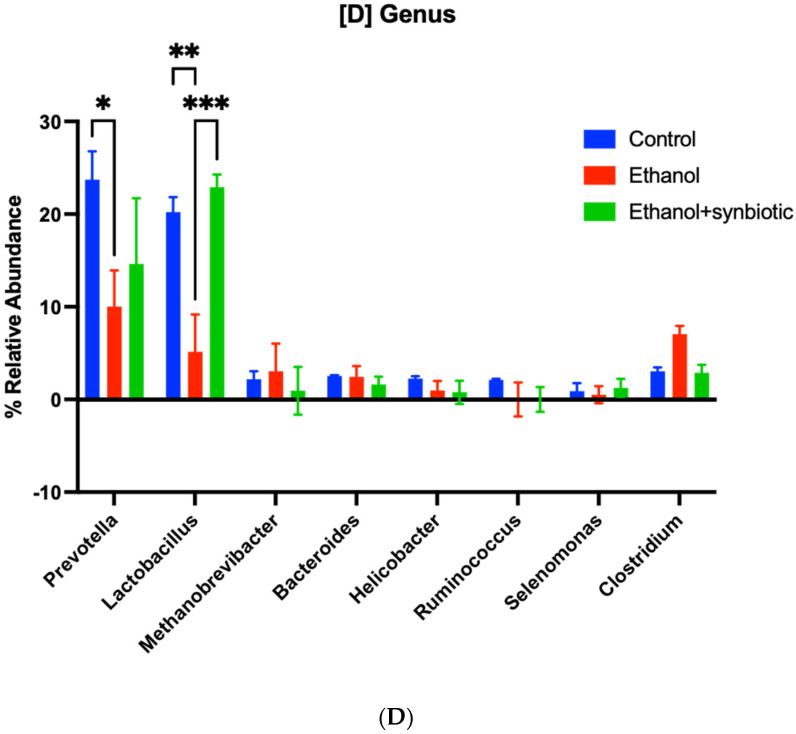
The microbiome of the acute alcoholic male rat model using control, ethanol, and ethanol-and-synbiotic (ES). Taxonomic profiles. Comparative representation of (**A**) Alpha Diversity: Red: Control; Green: Ethanol-and-synbiotic; Purple: Ethanol and (**B**) Beta Diversity, (**C**) phylum level, (**D**) genus level of selected genera among the groups, normalized by the control group. Applying a two-way ANOVA multiple comparison test between the mean of N = 3 to each group, significance is defined as a *p*-value < 0.05, * *p* < 0.05, ** *p* < 0.01, *** *p* < 0.001.

A Wistar male rat fecal sample (N = 3) was examined by the QIIME 2 tool for 16S rRNA analysis. As a result, after implementing the Lieber–DeCarli diet, the alpha diversity between the control, ethanol, probiotic, and synbiotic groups was examined to evaluate the gut microbiota associated with the acute alcohol model. After treatment, the ethanol group’s alpha diversity indices were negatively correlated and considerably lower than those of the control group (Figure 13A). Additionally, the ethanol-and-synbiotic treatment group had nearly identical diversity to the control group. However, although the changes were not substantial, the ethanol-and-synbiotic-treated group exhibited higher species richness and diversity indicators. The beta diversity analysis identified the ethanol-treated group from the standard control group (Figure 13B). Compared to the ethanol-and-synbiotic-treated, ethanol, and control groups, the fecal microbiota structure and composition differed considerably. *Firmicutes* were the most predominant phylum, accounting for 47.66%, 46.72%, and 58.19% of the gut microbiota in the control, ethanol, and ethanol-and-synbiotic groups, respectively. These were followed by *Bacteroidetes* (28.12%, 25.42%, and 30.70%, respectively) and *Proteobacteria* (3.47%, 16.40%, and 5.00%, respectively) (Figure 13C). However, in the ethanol-and-synbiotic and ethanol groups, the overgrowth of *Proteobacteria* and *Firmicutes was* not statistically significant. Additionally, abundances at the genus level in each group were significantly different. *Prevotella*, *Lactobacillus*, *[Prevotella]*, *Methanobrevibacter*, *Bacteroides*, *Helicobacter*, *Ruminococcin*, *Selenodonts*, and *Clostridium* increased in control, and ethanol-and-synbiotic groups, whereas *Prevotella* is most prevalent in the ethanol group. At the same time, the group that consumed ethanol-and-synbiotic had significantly more *Lactobacillus* (Figure 13D). These results suggest that ALD created an excess of proinflammatory pathobionts in a dysfunctional intestinal composition, which may have accelerated ALD.

## 4. Discussion

Alcohol promotes gut dysbiosis, lining, and microbial translocation [6,33]. Investigating the pathophysiology of ethanol-mediated gut barrier failure is essential from a biological and medical viewpoint since intestinal mucosal disruption is a critical mechanism in ALD progression. Despite intensive research over the past century, the molecular pathways underlying ethanol’s influence on gastrointestinal system functioning remain unknown. Consistently, excessive alcohol use has adverse effects on colon tissue, negatively influencing the host’s health. There are currently no dietary or pharmacological treatments available in the United States to prevent or cure ALD. Only alcohol abstinence and a liver transplant may prevent the development of ALD. Available treatments are personalized and limited [34]. One tailored therapy is probiotics such as *L. plantarum*, which decrease intestinal permeability caused by alcohol [19]. This is the first study to establish the preventative efficacy of AGE and *Lactobacillus rhamnosus* MTCC 1423 as a synbiotic against ALD. However, combining successful medicines may give multiple strategies for treating or preventing ethanol-induced gut dysbiosis, correlating to the findings of recent research [35,36] that drinking alcohol induces gut barrier impairment, permeability, inflammation, and ALD advancement. The present work assessed the permeability of rat colon and Caco-2 cells to determine the possible protective role of synbiotic therapy on alcohol-mediated intestinal mucosal barrier disruption. Results suggest that *Lactobacillus*, in conjunction with AGE, could inhibit gut barrier disruption significantly.

Caco-2 cells and rat intestinal epithelium demonstrated lower TEER (%) and higher intestinal leakage in the ethanol group, indicating that ethanol affects the colon in both in vitro and in vivo conditions [37,38]. Even in ethanol, treatment of probiotics lowered epithelial permeability, as observed in a previous study [39]. In addition, *Lactobacillus* supplementation reduces the intestinal barrier dysfunction triggered by alcohol [19]. Co-administration of *Lactobacillus rhamnosus* and AGE substantially prevented the decrease in TEER (%) even in the ethanol group, suggesting that synbiotic treatment may prevent gut barrier dysfunction. In addition, alcohol and/or its byproduct (acetaldehyde) affects TJ proteins, weakening the gut barrier function [40,41].

The connection between ZO-1 and occludin is essential for mucosal barrier function [42]. Occludin supports barrier function and structural integrity, while ZO-1 builds relationships with transmembrane proteins such as occludin [43,44]. Caco-2 monolayers exposed to 40 mM ethanol resulted in the development of disordered occludins and ZO-1, according to research [45]. Furthermore, acute alcohol consumption impairs intestinal membrane integrity by decreasing Tjs mRNA expression in mice [46]. Ethanol-induced rearrangement of TJ proteins is inhibited by probiotics such as *L. Plantarum* [19]. *A. muciniphila* reduced ethanol-mediated intestinal leakiness by increasing mucus thicknesses and expression of TJ proteins [47]. Pretreatment with *L. plantarum* ZLP001 inhibited ZO-1 and occludin expressions from lowering after enterotoxigenic Escherichia coli (ETEC) infection [48]. In Caco-2 monolayers and a mouse model of colitis, AGE prevented occludin, ZO-1, and microbe translocation [49]. AGE decreases TJ protein levels in the Caco-2 monolayers [50].

Recent studies suggest that AGE plus probiotics work synergistically to reduce IL-6 and TNF-a in the colon and serum, reducing inflammatory response significantly [51]. *L. plantarum* downregulated cytokine expression (TNF-a, IL-6) in the intestinal mucosa, inhibiting inflammation caused by ethanol [19]. Similarly, VSL#3 treatment reduces IL-1 expression in NOD mice’s intestinal mucosa [52]. By earlier results, the current examination showed substantially higher mRNA concentrations of IL-6 and TNF-a in ethanol-treated groups compared to control groups in both in vitro and in vivo experiments. Both studies showed that probiotics and AGE as a synbiotic significantly lowered TNF-a and IL-6 expression. A study reported that ethanol reduces IL-10 in the colon mucosa [42]. In a colorectal cancer model, genetically modified *Lactobacilli* strain combinations stimulated IL-10 [53]. As mentioned previously, alcohol consumption impairs intestinal permeability by inhibiting the TJ proteins’ expression. Restoring colonic permeability by ingesting a synbiotic may have more therapeutic value in preventing the course of ALD.

In the current work, both in vitro and in vivo ALD models demonstrated that synbiotics attenuate alcohol-induced TJ disruption. CYP2E1 may contribute to intestinal ethanol effects. It has been proven that CYP2E1 metabolites may be the principal source of ethanol-induced breakdown of the intestinal barrier as well as liver injury. CYP2E1 metabolism generates oxidative stress products such as MDA, which may generate reactive oxygen and nitrogen species that potentially damage the gut [14,50,54]. Alcohol-treated Caco-2 cells and animal studies exhibit increased CYP2E1 gene expression [13].

Along with CYP2E1, iNOS activation causes ROS production and alcohol-mediated mucosal barrier disruption [50]. The latest evidence revealed that garlic and probiotics, individually or in combination, significantly reduced oxidative stress in mice with liver diseases [55]. According to research, probiotics such as LC27 and LC67 can reduce gastrointestinal inflammation by inhibiting free radicals such as ROS [56]. The current results suggest that an increase in ROS expression levels in ALD models caused oxidative stress in the rat colon. A significant finding of this research was that a synbiotic administration suppresses CYP2E1, hence ameliorating colonic oxidative stress in ALD. Inflammation, as well as oxidative stress, may induce a leaky intestinal barrier. A leaky gut transgenic model showed intestinal inflammation [57]. Correlating with the research mentioned above our synbiotic administration significantly increased the expression of IL-10 a, and HPLC analysis of MDA resulted in the reduction of oxidative stress in both ALD models.

**Figure 14:**
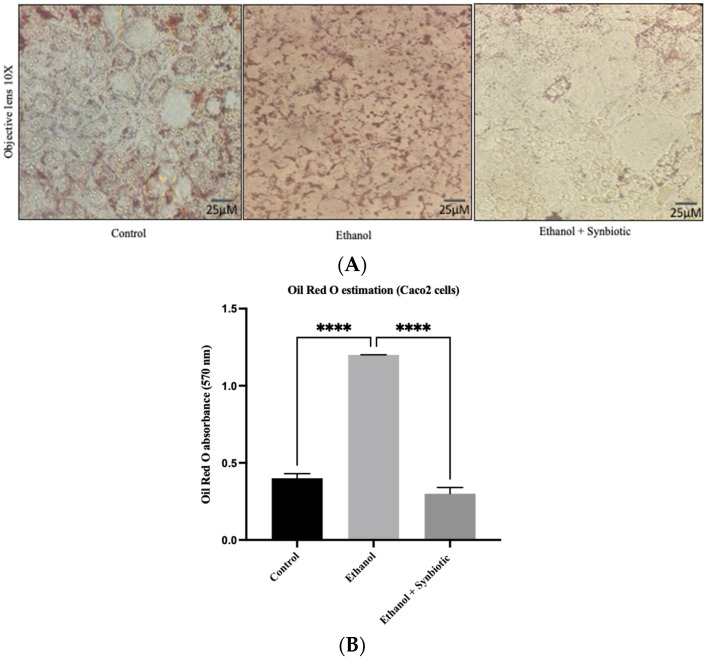
(**A**) Oil Red O-stained Caco-2 cells. Compared to the control group, the accumulation of lipids in Caco-2 cells increased in the ethanol group but was reduced significantly by synbiotic treatment at 100X. (**B**) Graphical representation of the Oil red O absorbance. The statistical analysis was calculated as mentioned in Section 2.10, **** *p* < 0.0001.

Previous research has established that ALD also alters lipid metabolism. Thus, the current work demonstrated that synbiotics trigger the pathway of AMPK and suppress the expression of SREBP-1c, consequently reducing ethanol-induced lipogenesis. Oil red O staining of the Caco-2 cells in Figure 14 of the colon tissue correlated with mRNA expression results and restored the barrier function, which is significant for the prevention of bacterial translocation from the gut to the portal vein, suggesting that the synbiotic administration may prevent the activation of the TLR4 pathway.

The gut microbiome affects ALD progression. Human diseases are usually triggered by *Bacteroides* spp., including *B. fragilis*, *Phocaeicola coprocola*, and Gram-negative anaerobes [58,59]. Probiotic administration decreased the density of *Bacteroides* in Wistar male rats using an acute alcohol paradigm, which is consistent with our study’s findings. Butyrate generating genus-like the *Clostridium* clusters, including *F. prausnitzii*, *L. eligens*, *and Clostridiales* spp., has positive impacts on the host compared to the pathogenic microbes [60,61]. According to a study on patients with alcoholic hepatitis, *L. rhamnosus* R0011 and *L. helveticus* R0052 restored the density of the bacteria that produce butyrate. Our investigation revealed identical outcomes by reintroducing the *Lactobacillus* species into the ALD model after administering a synbiotic treatment. It is also reported that *L. helveticus* changed enterohemorrhagic *E. coli* in the intestinal microenvironment, inhibiting the virulence associated with *E. coli* [62], and *L. rhamnosus* reduced *E. coli*-induced inflammation by lowering pro-inflammatory receptor expression [63]. The inverse relationship between pathogenic bacteria and probiotics may offer valuable data for designing probiotics-based therapies for ALD. Patients with cirrhosis had more significant proportions of *Proteobacteria* and *Fusobacteria* and a considerably decreased taxonomy richness of *Bacteroidetes* [64].

Bacteroidetes dominate the human gut microbiome. Therefore, its decline is commonly accompanied by a rise in Proteobacteria during inflammatory conditions [65,66]. An increase in the phylum Proteobacteria was observed in only ethanol-treated rats, with a distribution of 14.65% as compared to 3.48% in the control rats. These results corroborate previous findings, showing an increase in the phylum Proteobacteria in response to inflammatory conditions affecting the liver and gastrointestinal tract [67,68,69]. In contrast, upon treatment with the synbiotic in ethanol, the distribution of the phylum Proteobacteria significantly decreased to 5% compared to the ethanol-treated rats. This effect of synbiotic treatment resulting in a decrease in the Proteobacteria phylum might significantly ameliorate the ethanol-induced inflammatory response by the microbiota in the ALD rat model. Although it is well known that *Lactobacillus* can help the gut microbiota in several diseases, its application in the ALD model as a combination therapy has not yet been proven. A study on hypertension and cardiovascular diseases revealed that kyloic AGE improves microbial richness and diversity, with a marked increase in *Lactobacillus* and *Clostridia* species [70]. According to our knowledge, this is the first study to consider the positive impact of AGE as a synbiotic combination therapy for reestablishing gut flora. Relevant process taxonomic classification units identified in this study, and whose population fluctuations were very pertinent to cirrhosis and treatment, could be used as biomarkers in subsequent research. Our lack of clear mechanistic understanding of the interactions between specific pathogens responsible for dysbiosis and restoration of the gut microbiota has to be further investigated. Characterizing the roles and routes of the gut microbiome in ALD will require further research.

## 5. Conclusions

In the present study, in vitro and in vivo ALD models were analyzed to evaluate the relevance of oxidative stress and inflammation in ethanol-induced TJ disruption, colonic permeability, and fecal microbiota. According to the findings, synbiotic treatment successfully reduced oxidative stress and maintained TJ and gut microbiota in rats given ethanol. Furthermore, the current study aimed to advance pharmaceuticals that can restore commensal microbiota in alcohol-related gut imbalance. Based on this, the current regimen can be explored further to examine the effect of synbiotics on liver pathology to strengthen the realm of probiotic and combinational medicines as a treatment for ALD.

## Figures and Tables

**Table 1:** Human Colorectal adenocarcinoma Caco-2 cells primers list.

Sr. no.	Name of the Gene	Forward Sequence	Reverse Sequence
1	18S	ACGGAAGGGCACCACCAGGA	CACCACCACCCACGGAATCG
2	ZO-1	TATTATGGCACATCAGCACG	TGGGCAAACAGACCAAGC
3	Occludin	CTCGAGAAAGTGCTGAGTGCCTGGAC	AAGCTTTCGGTGACCAATTCACCTGA
4	CYP2E1	AGGGTACCATGTCTGCCCTCGGAGTGA	ACAATTTGAAAGCTTGTTTGAAAGCGG
5	NOX	GTACAAATTCCAGTGTGCAGACCAC	GTACAAATTCCAGTGTGCAGACCAC
6	TNF-α	CCCTCACACTCAGATCATCTTCT	GCTACGACGTGGGCTACAG
7	IL-6	GACAACTTTGGCATTGTGG	ATGCAGGGATGATGTTCTG
8	IL-10	ACTGCTAACCGACTCCTTA	TAAGGAGTCGGTTAGCAGT

**Table 2:** List of Rat colon primers.

Sr. no.	Name of the Gene	Forward Sequence	Reverse Sequence
1	18S	ACGGAAGGGCACCACCAGGA	CACCACCACCCACGGAATCG
2	ZO-1	TATTATGGCACATCAGCACG	TGGGCAAACAGACCAAGC
3	Occludin	CTCGAGAAAGTGCTGAGTGCCTGGAC	AAGCTTTCGGTGACCAATTCACCTGA
4	CYP2E1	AGGGTACCATGTCTGCCCTCGGAGTGA	ACAATTTGAAAGCTTGTTTGAAAGCGG
5	NOX	GTACAAATTCCAGTGTGCAGACCAC	GTACAAATTCCAGTGTGCAGACCAC
6	TNF-*α*	CCCTCACACTCAGATCATCTTCT	GCTACGACGTGGGCTACAG
7	IL-6	GACAACTTTGGCATTGTGG	ATGCAGGGATGATGTTCTG
8	IL-10	ACTGCTAACCGACTCCTTA	TAAGGAGTCGGTTAGCAGT

## Data Availability

The authors confirm that the data supporting the findings of this study are available within the manuscript and uploaded jpeg files.
